# The Puerperal Diseases—Barker

**Published:** 1874-03

**Authors:** 


The Puerperal Diseases. *Clinical lectures delivered at Bellevue Hospital.* By Fordyce Barker, M.D., Clinical Professor of Midwifery and the Diseases of Women, in the Bellevue Hospital Medical College, etc. New York: D. Appleton & Co., 1874. Octavo, cloth, pp. 526. Sold in Atlanta by J. J. & S. P. Richards. Review next month.

